# Towards the quantized anomalous Hall effect in AlO_x_-capped MnBi_2_Te_4_

**DOI:** 10.1038/s41467-025-57039-7

**Published:** 2025-02-18

**Authors:** Yongqian Wang, Bohan Fu, Yongchao Wang, Zichen Lian, Shuai Yang, Yaoxin Li, Liangcai Xu, Zhiting Gao, Xiaotian Yang, Wenbo Wang, Wanjun Jiang, Jinsong Zhang, Yayu Wang, Chang Liu

**Affiliations:** 1 School of Physics, Renmin University of China, Beijing, China; 2 Key Laboratory of Quantum State Construction and Manipulation (Ministry of Education), Renmin University of China, Beijing, China; 3 State Key Laboratory of Low Dimensional Quantum Physics, Department of Physics, Tsinghua University, Beijing, China; 4 Beijing Academy of Quantum Information Sciences, Beijing, China; 5 School of Physical Science and Technology, ShanghaiTech Laboratory for Topological Physics, ShanghaiTech University, Shanghai, China; 6 Frontier Science Center for Quantum Information, Beijing, China; 7 Hefei National Laboratory, Hefei, China; 8 New Cornerstone Science Laboratory, Frontier Science Center for Quantum Information, Beijing, P. R. China

**Keywords:** Topological insulators, Surfaces, interfaces and thin films

## Abstract

The quantum anomalous Hall effect in layered antiferromagnet MnBi_2_Te_4_ harbors a rich interplay between magnetism and topology, holding a significant promise for low-power electronic devices and topological antiferromagnetic spintronics. In recent years, MnBi_2_Te_4_ has garnered considerable attention as the only known material to exhibit the antiferromagnetic quantum anomalous Hall effect. However, this field faces significant challenges as the quantization at zero magnetic field depending critically on fabricating high-quality devices. In this article, we introduce a straightforward yet effective method to mitigate the detrimental effect of the standard fabrication on MnBi_2_Te_4_ by depositing an AlO_x_ layer on the surface before fabrication. Optical contrast and magnetotransport measurements on over 50 MnBi_2_Te_4_ demonstrate that AlO_x_ can effectively preserve the pristine states of the devices. Surprisingly, we find this simple method can significantly enhance the anomalous Hall effect towards quantization, which resolves a longstanding challenge in the field of MnBi_2_Te_4_. Scaling relation analysis further reveals the intrinsic mechanism of anomalous Hall effect dominated by Berry curvature at various magnetic configuration. By tuning the gate voltage, we uncover a gate independent magnetism in odd-layer MnBi_2_Te_4_ devices. Our experiments not only pave the way for the fabrication of high-quality dissipationless transport devices, but also advance the investigation of exotic topological quantum phenomena in 2D materials.

## Abstract (web summary)

The challenges in reliably reproducing the quantum anomalous Hall effect have emerged as a major bottleneck for MnBi_2_Te_4_. Here, the authors develop a fabrication method to address this, paving the way for the fabrication of high-quality dissipationless topological transport devices.

## Introduction

**Fig. 1:**
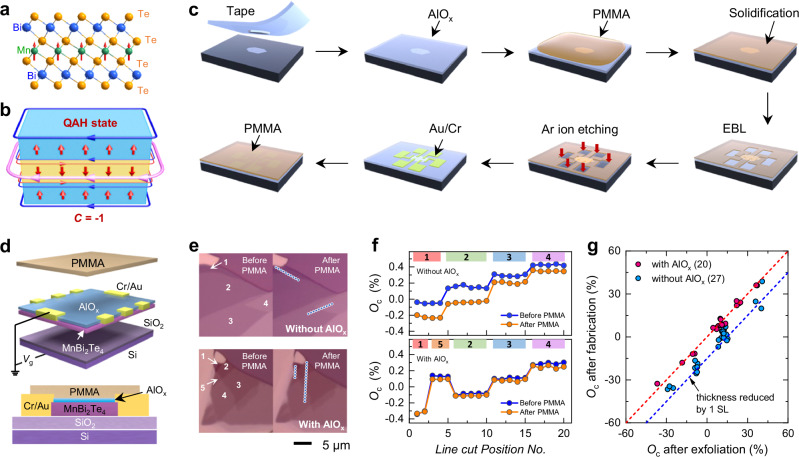
Fabrication and optical contrast characterizations of few-layer MnBi_2_Te_4_ flakes. **a** Crystal structure of MnBi_2_Te_4_. **b** Schematic of the QAH effect in an odd-SL MnBi_2_Te_4_. The red arrows in **a** and **b** represent the magnetic moments of Mn in each layer. **c** Illustration of the device fabrication process. This method is developed based on the standard EBL process. By simply depositing a thin layer of AlO_x_ on the MnBi_2_Te_4_ surface, the PMMA resist is isolated from the top surface. The high insulation and compactness of AlO_x_ make it possible to fabricate Hall bar patterns while protecting the sample from chemical reagents. **d** Front and side views of a transport device. **e** Optical images of MnBi_2_Te_4_ flakes exfoliated from the same crystal. The top (bottom) panel compares the color change of MnBi_2_Te_4_ flakes without (with) AlO_x_ capping before and after contact with PMMA, respectively. **f** Variation of *O*_c_ for selected spots along the line traces in flakes of different thicknesses in (**e**). *O*_c_ is defined as (*I*_flake_ - *I*_substrate_)/*I*_substrate_, where *I*_flake_ and *I*_substrate_ are the intensity of MnBi_2_Te_4_ and substrate, respectively. **g** Statistical analysis of *O*_c_ of 47 MnBi_2_Te_4_ flakes with (red) and without (blue) AlO_x_ capping layer. Different data points represent the *O*_c_ of different flakes after mechanical exfoliation and after contact with PMMA, respectively. The red and blue dashed line denote the *O*_c_ reduction by 0 and 20%, respectively, corresponding to no change and a decrease of effective thickness by one layer. The variability of the degree of *O*_c_ reduction among different flakes does not stem from measurement errors but rather arises from the inhomogeneities and sample quality fluctuations within the bulk crystal.

Magnetic topological materials have emerged as a frontier in condensed matter physics, providing promising platforms for exploring exotic quantum phenomena and applications in topological spintronics^1–3^. For uncovering novel topological physics, successful fabrication of high-quality devices with quantized transport is prerequisite. As the first identified material possessing van der Waals characteristics, intrinsic magnetism, and nontrivial band topology simultaneously, MnBi_2_Te_4_ not only exhibits rich novel quantized phenomena when exfoliated down to few-layer limit^4–6^, but is also considered capable of addressing the disorder issue that is prevalent in magnetically doped topological insulators (TIs)^7^. The bulk crystal of MnBi_2_Te_4_ can be regarded as a stacking of Te–Bi–Te–Mn–Te–Bi–Te septuple layer (SL) along *z*-direction (Fig. 1a). A-type antiferromagnetic (AFM) structure with interlayer AFM order and intralayer ferromagnetic (FM) order forms below the Néel temperature (*T*_N_) ~ 25 K. When interacting with band topology, this layer-dependent magnetic ordering can give rise to a rich variety of topological quantum states and exotic magnetoelectric response^8–15^. In odd-SL MnBi_2_Te_4_ film, the gapped Dirac topological surface states due to the parallel surface magnetizations drive the system into the quantum anomalous Hall (QAH) state with 1D dissipationless chiral edge state transport^8^ (Fig. 1b). This manifests as a quantized Hall conductivity *σ*_xy_ = *Ce*^2^/*h* at zero-magnetic field (*μ*_0_*H* = 0), where *C* represents the Chern number, *e* is the electron charge, and *h* is the Planck constant. In even-SL MnBi_2_Te_4_, the opposite surface magnetizations result in vanishing *σ*_xy_ and lead to the axion insulator state characterized by a zero plateau at *μ*_0_*H* (ref. ^10^). Recent progresses in MnBi_2_Te_4_ have unveiled a plethora of novel topological phenomena, including the Möbius insulator^16^, layer Hall effect^11^, axion optical induction^17^, and quantum metric nonlinear transport^18,19^.

Although the QAH effect and axion insulator states have been observed in 5- and 6-SL MnBi_2_Te_4_, the temperature (*T*) below which quantization is realized remains much lower than its *T*_N_. A more formidable challenge arises from the exceptionally low yield of MnBi_2_Te_4_ film exhibiting quantized transport. Over the past five years, neither perfectly quantized^9,12,13,15,20–22^ nor zero plateau^12,17–19,22^ at *μ*_0_*H* = 0 can be consistently reproduced. The lack of quantization not only obstructs the discovery of new phenomena but also complicates the interpretation of available data. Possible reasons include various structural defects or impurity phases^23–27^, instability of surface electronic structures^28–31^, and weakened surface out-of-plane magnetic anisotropy (MA)^31–33^. Our recent studies combining optical contrast (*O*_c_), transport, magneto-optical Kerr effect (MOKE) measurements revealed a substantial impact of fabrication on the properties of MnBi_2_Te_4_ devices^22^. The contact with photoresist not only reduces the *O*_c_ value during the fabrication process, but may also results in mismatched even-odd-layer-dependent magnetotransport^22^. The mechanism likely originates from the formation of a dead insulating layer on the MnBi_2_Te_4_ surface, which is caused by the change of surface band structure^30,34,35^. Over the past five years, developing a low-damage fabrication method to reproduce the QAH effect has become one of the most pressing tasks in the field of magnetic topological quantum materials and devices.

In this work, we optimize the fabrication process by depositing an AlO_x_ protective layer on MnBi_2_Te_4_ top surface prior to the standard electron beam lithography (EBL). To ensure a good ohmic contact between the MnBi_2_Te_4_ and electrode, we implement an additional Ar ion etching step to selectively remove the AlO_x_ at the electrode areas after the lithography process. By employing this method, we can fabricate transport devices with good electrical contact while using an insulating AlO_x_ layer to fully isolate the photoresist Polymethyl Methacrylate (PMMA). Through optical measurement on a series of MnBi_2_Te_4_ thin flakes, we find that the fabrication issue induced by PMMA photoresist is largely mitigated. Most importantly, this simple idea overcomes the bottlenecks in the field of topological quantum materials over the past five years. We not only achieve the QAH effect in multiple MnBi_2_Te_4_ devices, but also reveal the key factors influencing the zero-magnetic-field quantization. Our work introduces a simple yet effective method for fabricating high-quality transport devices, paving the way for realizing the QAH effect and exploring more exotic topological quantum phenomena.

## Results

### Device fabrication and optical contrast

We got inspiration from previous experiments where those MnBi_2_Te_4_ devices exhibiting large anomalous Hall (AH) effect often had Al_2_O_3_ on the bottom of the flakes^8,21,36^, either as a substrate or a supporting layer. This implies that the contact with Al_2_O_3_ may help to improve the quality of MnBi_2_Te_4_ device. Combined with our recent finding of the detrimental effect of fabrication on the top surface of MnBi_2_Te_4_, we come up with a straightforward yet effective idea that by depositing an AlO_x_ layer on top of MnBi_2_Te_4_ to achieve the QAH effect. Figure 1c shows the schematic of the fabrication process. First, we transferred thick MnBi_2_Te_4_ flakes from a bulk crystal to the substrate using a Scotch tape. We then employed the mechanical exfoliation method to obtain the flakes with target thickness. The one-to-one correspondence between *O*_c_ and thickness allows the rapid determination of SL number by optical method^11^. Subsequently, a 3-nm AlO_x_ layer was deposited by thermal evaporation. We then adopted the standard EBL process to expose the designed Hall bar patterns. Next, the AlO_x_ layer above the designed electrode areas was etched away by Ar ion etching, followed by the deposition of Cr/Au electrodes. To ensure the charge transport is not affected by the etching process in the contact region, we assessed the MnBi_2_Te_4_ thickness in the etched region by *O*_c_ and atomic force microscopy measurements (see Supplementary Figs. 1 and 2). Finally, a PMMA layer was coated for further protection. The details of the fabrication are described in the Methods section. Compared to the Al_2_O_3_-assisted exfoliation and stencil mask method^8^, our method is more straightforward and is based on the standard EBL process, which enables the fabrication of specific nano-devices with reduced sample size. Furthermore, since the AlO_x_ is on the top surface, it not only offers an effective protection, but also can be used as a dielectric layer for top gate controllability.

Figure 1d depicts a schematic of a Hall bar device covered with AlO_x_ capping layer and its cross-sectional view. To investigate the influence of AlO_x_ on MnBi_2_Te_4_, we first compare the optical properties of MnBi_2_Te_4_ flakes with varied thicknesses, which were exfoliated from the same single crystal (Supplementary Fig. 3 for the *O*_c_ change in each step). The optical images in Fig. 1e clearly suggest that for flakes without AlO_x_ (up panel), the colors of all four regions change significantly before and after contact with PMMA. In contrast, those regimes with AlO_x_ (down panel) do not exhibit noticeable change during the same process. To further explore the effect of AlO_x_ quantitatively, we extract their *O*_c_ values of the selected spots along the line traces in Fig. 1e and compare their variations directly. As shown in Fig. 1f, significant reductions of *O*_c_ in all the four areas without AlO_x_ are observed. In contrast, *O*_c_ remains nearly unchanged for the five areas with AlO_x_ capping layers. To eliminate the influences of device quality fluctuations on our observation, we compare the *O*_c_ values of 47 MnBi_2_Te_4_ exfoliated from the same crystal (Fig. 1g). Different data points represent the *O*_c_ of different flakes after exfoliation and after contact with PMMA. The distribution of *O*_c_ falls well into two parts (red and blue dots). According to the one-to-one correspondence between *O*_c_ and thickness^11^, the decrease of *O*_c_ indicates the reduction of effective thickness during fabrication. The red and blue dashed lines represent the *O*_c_ reduction by 0 and 20 %, respectively, which corresponds to unchanged thickness and a decrease of thickness by one SL. For the flakes without AlO_x_ layer, regardless of their initial *O*_c_ values, most of the samples display a pronounced decrease in *O*_c_ after fabrication. Notably, the degree of *O*_c_ change exhibits certain variability among different samples. This behavior does not stem from measurement errors but rather originates from the inhomogeneities and sample quality fluctuations within the MnBi_2_Te_4_ crystal. Such sample-dependent sensitivity to fabrication has also been observed in previous experiments^22^. These results clearly demonstrate that AlO_x_ can effectively mitigates the damages caused by PMMA^22^.

### Statistical analysis of the influence of AlO_x_ on transport properties

In magnetic topological systems, the AH effect typically results from three mechanisms: intrinsic Berry curvature Ω(**k**), skew-scattering, and side-jump. In the transport of MnBi_2_Te_4_, due to defects or impurity phases, all the three mechanisms could contribute to the AH effect^20^. However, in an ideal quantized Hall system, the transverse transport should be dominated by Ω(**k**) in momentum (**k**) space^37^. Theoretically, *σ*_xy_ can be calculated by integrating Ω over **k**, as expressed by:

$${\sigma }_{{xy}}=-\frac{{e}^{2}}{2\pi h}\int \Omega \left({{\bf{k}}}\right){d}^{2}{{\bf{k}}}$$

**Fig. 2:**
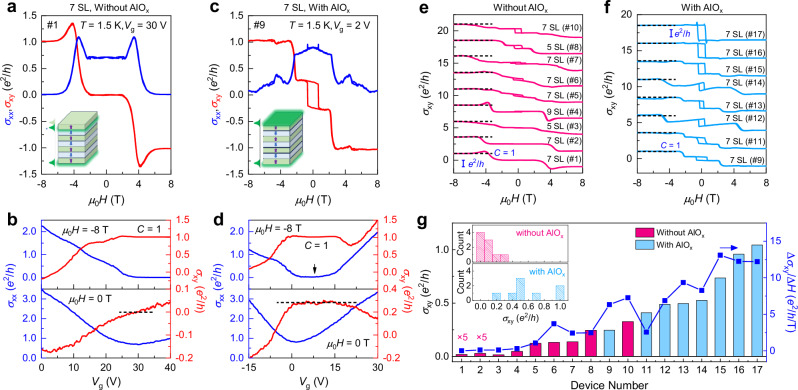
Comparison of transport behaviors for devices obtained by different fabrication methods. **a** Transport behaviors at CNP for a 7-SL device without AlO_x_ covering layer. Due to fabrication effects, the surface state shifts down to the second SL (inset), and the hysteresis of *σ*_xy_ near zero field almost disappears. **b** Variation of *σ*_xy_ and *σ*_xx_ as a function of *V*_g_ at *μ*_0_*H* = −8 T and 0, respectively. The black dashed line represents the *V*_g_ window where *σ*_xy_ plateaus coexist under zero and high *μ*_0_*H* conditions, respectively. **c** Transport behavior of a 7-SL device exfoliated from a MnBi_2_Te_4_ flake on the same tape, but with an AlO_x_ layer deposited during the fabrication process. The large hysteresis indicates excellent protection of device performance. The inset illustrates that the topological surface state remains predominantly distributed on the outermost surface due to the protection of AlO_x_. **d** In the same *V*_g_ range of high field Chern insulator state (marked by black dashed line), *σ*_xy_ at *μ*_0_*H* = 0 exhibits a broad plateau during sweeping *V*_g_. **e**, **f** *μ*_0_*H*-dependent *σ*_xy_ at *T* = 1.5 K for 17 odd-SL devices. The only difference during their fabrication lies in whether the surface was deposited with AlO_x_. All the devices exhibit quantized *σ*_xy_ at high *μ*_0_*H*, as marked by the black dashed lines. **g** Summarized *σ*_xy_ at *μ*_0_*H* = 0 and ∆*σ*_xy_/∆*H* values at the plateau transition for the 17 devices. Devices with AlO_x_ capping layer (blue) generally show a larger AH effect than those without AlO_x_ (red). The inset displays the histograms of AH effect distribution of the 17 devices. The size of each bin on the *σ*_xy_-axis is 0.1 *e*^2^/*h*.

When the Fermi level (*E*_F_) is tuned into the magnetic exchange gap, the integral of Ω equals the *C* number multiplied by 2π, resulting in the quantization of *σ*_xy_ at *e*^2^/*h*. To investigate the influence of AlO_x_ on the intrinsic AH effect, we measured the transport properties of 17 odd-SL MnBi_2_Te_4_ devices. All the data shown in the main text was obtained at the charge neutral point (CNP) unless otherwise specified. Prior to this, we measured the current–voltage curve of the AlO_x_ layer to exclude its contribution to transport (Supplementary Fig. 4). Figure 2a–d shows the *μ*_0_*H* dependence of *σ*_xy_ and *σ*_xx_ for two 7-SL devices exfoliated from the same thick flake on the same tape. Both devices show quantized *σ*_xy_ at the high *μ*_0_*H* Chern insulator state (*C* = −1). However, their AH effects at zero field exhibit dramatically different behaviors. For device #1 without AlO_x_ (Fig. 2a), *σ*_xy_ almost vanishes at the low *μ*_0_*H* AFM regime. Such behavior is consistent with our previous observation that fabrication process can damage the top surface, leading to a reduction of effective thickness by one SL^22^. However, for the device #9 fabricated by the current method, a large *σ*_xy_ accompanied with a square-shaped hysteresis is observed. The insets show the schematic distribution of the topological surface states wave functions (green) for devices without and with AlO_x_. To further demonstrate the influence of AlO_x_ on transport more clearly, we display the gate voltage (*V*_g_) dependent *σ*_xy_ and *σ*_xx_ at *μ*_0_*H* = −8 T and 0 for the two devices, as displayed in Fig. 2b, d. For device #1, *σ*_xy_ smoothly crosses zero with increasing *V*_g_, indicating a reduced gap size during the fabrication process^22^. In contrast, device #9 shows a wide plateau in *σ*_xy_ in the same *V*_g_ range of the Chern insulator, indicating an incipient QAH state in the AFM state.

For 2D materials, the transport properties of thin flakes are inevitably influenced by the fluctuations of device qualities. Previous experiments have suggested that MnBi_2_Te_4_ exhibits sample-dependent properties, even for flakes prepared from the same crystal^8,24,25^. Therefore, it is challenging to expect any fabrication method to guarantee the production of perfect QAH devices with 100 % certainty. To demonstrate that the enhancement of the AH effect arises from AlO_x_, we studied the transport properties of 17 odd-number-SL MnBi_2_Te_4_ devices, with the data presented in Fig. 2e, f. These devices were numbered based on the increasing order of their *σ*_xy_ at *μ*_0_*H* = 0. Apart from devices #2, #4, #6, and #8 that were acquired from crystal #1, all the other 13 devices were obtained from crystal #2. All the devices exhibit *σ*_xy_ = *e*^2^/*h* at the high *μ*_0_*H* Chern insulator state, indicating the overall high quality of our devices. However, these devices show dramatically different behaviors in their AH effect. Remarkably, the 9 devices without AlO_x_ layer exhibit small *σ*_xy_ at *μ*_0_*H* = 0. And some devices even exhibit almost indiscernible hysteresis. In sharp contrast, all the other 8 devices with AlO_x_ capping layer manifest large *σ*_xy_ and square-shaped hysteresis, with two devices almost quantized at *e*^2^/*h*. Figure 2g summarizes the *σ*_xy_ at *μ*_0_*H* = 0 of these devices. To largely avoid any artificial trend, we adopted the strategy in previous statistical studies of MnBi_2_Te_4_ crystals^25^ by sorting the *σ*_xy_ from smallest to largest. In the inset of Fig. 2g, we also present the histograms of their *σ*_xy_ distribution. The size of each bin on the *σ*_xy_-axis is 0.1 *e*^2^/*h*. For the devices without AlO_x_, their *σ*_xy_ values are distributed within the range of 0 to 0.3 *e*^2^/*h*. In contrast, for those devices with AlO_x_, their *σ*_xy_ distribution displays a clear shift towards higher values. Notably, despite not all devices with AlO_x_ exhibit the QAH effect, their AH effects have already surpassed the values for most MnBi_2_Te_4_ devices in literatures^9,11–13,20,22^. In Fig. 2g, we also summarized the values of ∆*σ*_xy_/∆*H* of the 17 devices (blue points), which represent the sharpness of magnetic transition. The variation of ∆*σ*_xy_/∆*H* aligns with the trend of *σ*_xy_. These results undoubtedly demonstrate that AlO_x_ plays a significant role in enhancing the AH effect.

**Fig. 3:**
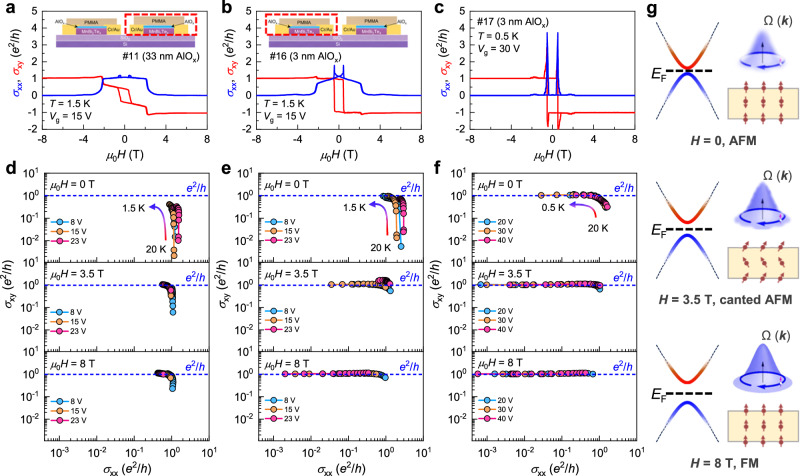
Scaling relation between *σ*_xy_ and *σ*_xx_ of the intrinsic AH effect. **a**–**c** *μ*_0_*H*-dependent *σ*_xy_ and *σ*_xx_ for three 7-SL devices exfoliated from the same crystal. Devices #11 and #16 were obtained simultaneously in one cleaving process on the same substrate, with the former one undergoing an extra AlO_x_ deposition process, having a thickness of 33 nm. **d**–**f** Evolution of *σ*_xy_ with *σ*_xx_ during the cooling process. With the formation of AFM order as lowering *T*s, the scaling relation between *σ*_xy_ and *σ*_xx_ at different *V*_g_s gradually collapses into one single curve, and *σ*_xy_ saturates at *e*^2^/*h*. The *σ*_xx_ independent behavior reflects the typical Berry curvature-dominated mechanism of the AH effect. **g** Schematics of the distribution of Berry curvature. From top to bottom, as the AFM order is tuned to the FM order, the out-of-plane component of the total magnetization is enhanced. As a result, the exchange gap increases, and the Berry curvature exhibits greater robustness against thermal fluctuations.

The fabrication of high-quality devices enables us to compare the influences of magnetic properties on transport. Figure 3a–c shows the *μ*_0_*H*-dependent *σ*_xx_ and *σ*_xy_ for three devices obtained from the same crystal. Fortunately, for devices #11 and #16, they were obtained on the same substrate during one cleaving process. It enables us to further explore the influences of AlO_x_ on MnBi_2_Te_4_ flake while preserving the consistency of the devices. Overall, the three devices manifest consistent transport behaviors, with their main differences being the values of *σ*_xx_ and *σ*_xy_ at *μ*_0_*H* = 0. However, the sharpness of the plateau transition, which reflects the magnetic flipping process, differs dramatically. For device #11, the *σ*_xy_ and *σ*_xx_ at *μ*_0_*H* = 0 are 0.5 and 1.1 *e*^2^/*h*, respectively, and the transition is relatively gentle. For device #16, although the value of *σ*_xx_ does not change, *σ*_xy_ is significantly improved to 0.96 *e*^2^/*h*, comparable to the value in previous report^8^. In addition, the plateau transition is also sharper than that of device #11. Device #17 completely enters the QAH state, with *σ*_xy_ reaching *e*^2^/*h* and *σ*_xx_ dropping to 0. Because the QAH effect in magnetic TIs originates from the exchange field between local moments and electron spins^38^. The out-of-plane magnetic order plays a crucial role in the *μ*_0_*H*-dependent transport behaviors. Therefore, the improved quantization along with the sharp *σ*_xy_ transition suggests that device #17 is likely to have a stronger out-of-plane MA.

The scaling relation between *σ*_xy_ and *σ*_xx_ may further help us understand the role of AlO_x_ in enhancing the QAH effect. Figure 3d–f displays the variation of *σ*_xy_ as a function of *σ*_xx_ during the cooling process under different *μ*_0_*H* and *V*_g_s. As the AFM order strengthens at low *T*s, *σ*_xy_ begins to exhibit behavior independent of *σ*_xx_ and gradually approaches quantization, which is of typical the scaling behavior of the intrinsic AH effect dominated by Ω(**k**) (ref. ^37^). Upon increasing *μ*_0_*H*, the device undergoes AFM, canted AFM, and finally enters FM state, accompanied by *σ*_xy_ saturating at *e*^2^/*h* at higher *T*s. For device #11 with relatively weaker out-of-plane magnetic order, the exchange gap is expected to be smaller in the AFM state, thermal fluctuations could more easily smear out the role of Ω(**k**) (top in Fig. 3g). Therefore, complete quantization appears only when all moments are parallelly aligned because the gap is overall positively correlated with magnetization. However, for the device #17 with stronger out-of-plane order, the larger gap allows for *σ*_xy_ reaching quantization even in the AFM state despite a small net moment. The influence of different magnetic configurations on exchange gap and Ω(**k**) is illustrated in Fig. 3g. Ω(**k**) can be interpreted as an effective *μ*_0_*H* in **k** space acting on electrons. The red and blue represent the distribution of Ω(***k***) of opposite sign in conduction and valence band, respectively.

### Gate voltage-independent magnetism

**Fig. 4:**
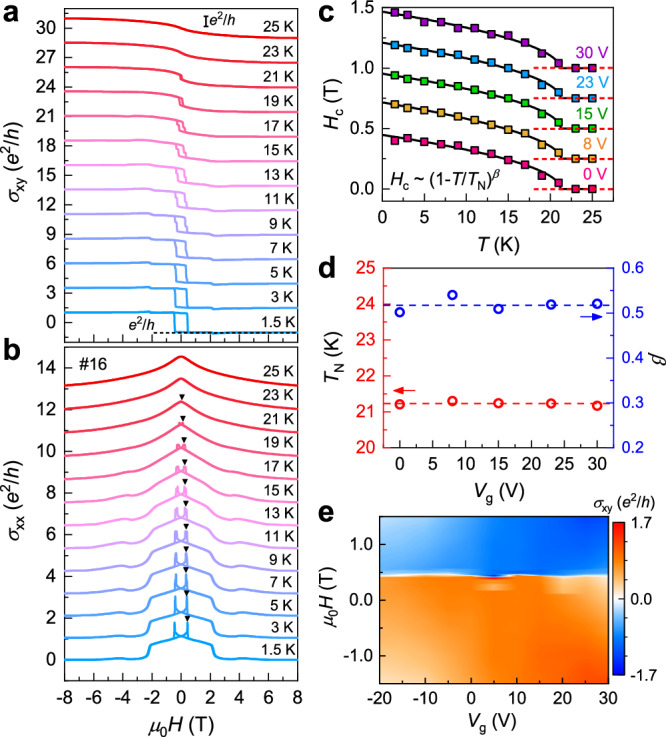
Transport and magnetic properties tuned by *V*_g_. **a**, **b** *μ*_0_*H*-dependent *σ*_xy_ and *σ*_xx_ at the CNP for device #16 at various *T*s. The hysteresis and the double peak in *σ*_xx_ disappear at around *T* = 21 K. The black triangles mark the position of *H*_c_ at different *T*s. **c** *H*_c_ extracted from the field sweep data as a function of *T* at varied *V*_g_s. The solid squares are the data points. The black lines are the data fittings in the form of ~(1 − *T*/*T*_N_)^*β*^. The red dashed lines represent the position of *H*_c_ = 0 for each curve. **d** Summarized *T*_N_ and *β* from the fittings as a function of *V*_g_. The blue and red dashed lines represent the average positions of the *β* and *T*_N_ values for different *V*_g_s. *T*_N_ and *β* are found to be ~21.2 K and 0.52, respectively, both of which are independent of *V*_g_. **e** Colormap of *σ*_xy_ in the parameter space of *μ*_0_*H* and *V*_g_. The boundary between blue and orange region marks the *V*_g_-independent *H*_c_.

Next, we investigate the influence of *V*_g_ on the magnetic properties of MnBi_2_Te_4_ devices. Previous studies have revealed different *V*_g_-dependent magnetism on magnetically doped TIs^39–41^. The electrical control of van der Waals magnetism has also attracted wide attention. As the first layered topological antiferromagnet, it remains unclear whether *V*_g_ can exert similar effects. Figure 4a, b displays the *μ*_0_*H*-dependent *σ*_xy_ and *σ*_xx_ for device #16 at various *T*s. The hysteresis vanishes at around *T* = 21 K, accompanied by the disappearance of *σ*_xx_ peaks. To quantitatively investigate the changes in the AFM state, we extract the coercive field (*H*_c_) values at different *V*_g_s and plot them as a function of *T* with an offset of 0.25 T (Fig. 4c). The *H*_c_ dependence of *T* can be well described by the power law ~(1 − *T*/*T*_N_)^*β*^, where *β* represents the critical exponent. We notice that *V*_g_ has almost negligible effect on the AFM order. *T*_N_ remains a constant at ~21.3 K and *β* maintains at ~0.52. Similar results were also observed in previous neutron diffraction on MnBi_2_Te_4_ bulk crystal and reflectance magneto-circular dichroism (RMCD) measurement on exfoliated thin flakes^14,42^. Our experiments further point that this critical behavior cannot be tuned by a bottom *V*_g_. Figure 4e shows the colormap of *σ*_xy_ as a function of *V*_g_ and *μ*_0_*H*. It clearly shows that *H*_c_ is independent of *V*_g_, further supporting the *V*_g_-independent magnetism in odd-SL MnBi_2_Te_4_ device. Reproducible results obtained from another two 7-SL MnBi_2_Te_4_ with and without AlO_x_ capping layer are documented in Supplementary Figs. 5 and 6.

## Discussion

Finally, we discuss the possible mechanisms underlying the enhancement of AH effect. In our previous research, we found that the coating of PMMA during the EBL process reduces the *O*_c_ of MnBi_2_Te_4_, leading to a reduction of effective thickness^22^. AlO_x_ serves as an effective barrier by isolating the surface from direct contact with the resist, thus providing a substantial protection for MnBi_2_Te_4_. However, in the history of 2D materials, the most widely used and effective capping layer for protection is *h*-BN, rather than AlO_x_. In fact, previous experiments have suggested that the oxidation process may alter the intrinsic properties of MnBi_2_Te film^28^, therefore, using an AlO_x_ capping layer to protect MnBi_2_Te is unconventional (Supplementary Fig. 7 for the aging effect). Furthermore, beyond employing a protective layer, a shadow mask method can also be employed to avoid direct contact with PMMA resist. However, many experiments have demonstrated that even employing these methods^13,18,20,21^, the AH effect remains non-quantized. Interestingly, a comparison of recent transport experiments in MnBi_2_Te_4_ reveals that regardless of the different device preparation or electrode deposition methods, all MnBi_2_Te_4_ devices exhibiting pronounced AH effect have one surface in contact with Al_2_O_3_ (refs. ^8,21,36^). In these experiments, Al_2_O_3_ is positioned under MnBi_2_Te_4_ and does not provide any protection to the top surface. Hence, the simple protective role is insufficient to explain the close correlation between large AH effect and AlO_x_ in current experiments. It naturally raises the question of whether AlO_x_ may play an additional role beyond protection.

Our scaling relation studies imply that the enhancement of perpendicular magnetic order may be crucial for the QAH effect. Based on our experimental data and the results in previous studies, we discuss the potential additional roles that AlO_x_ may play. A conceivable scenario is the electric field enhanced magnetism at the AlO_x_/MnBi_2_Te_4_ interface^43,44^. However, this scenario can be largely excluded because our *V*_g_-dependent experiments demonstrate that *V*_g_ has a small influence on magnetism. Another possible scenario is the enhanced perpendicular magnetic anisotropy (PMA) by AlO_x_. In spintronics, many experiments have demonstrated that depositing amorphous oxide (such as AlO_x_, MgO, TaO_x_, HfO_x_) can substantially increase the interfacial PMA at the interface between oxides and magnetic materials^45–47^. Therefore, it is naturally expected that the AlO_x_ layer strengthens the interfacial PMA of MnBi_2_Te_4_, which in turn enhances the AH effect. In fact, there has been theoretical calculations suggesting that the MA in monolayer MnBi_2_Te_4_ is weak due to the weak *p*-*d* hybridization between Mn and Te (ref. ^48^). Later, inelastic neutron scattering pointed that for MnBi_2_Te_4_ crystals, the MA is enhanced by the interlayer two-ion anisotropy^32^. However, due to the absence of neighboring layers, the MA of the surface is still weak. These results naturally explain why, in all current MnBi_2_Te_4_ experiments exhibiting a large AH effect, the device must have at least one surface in contact with AlO_x_.

To validate our conjectures, we conducted cryogenic magnetic force microscopy (MFM) measurement to directly visualize the magnetic properties across different regions of the same 7-SL MnBi_2_Te_4_. As anticipated, the region with AlO_x_ manifests a much stronger magnetic signal compared to the region without AlO_x_ (Supplementary Fig. 8). Moreover, to further explore the influence of AlO_x_ on QAH effect, we compared the magnetic hysteresis loops of two fully quantized devices with single-sided and double-sided AlO_x_ contacts. Interestingly, the device with both top and bottom surfaces in contact with AlO_x_ shows a larger *H*_c_ (see Supplementary Fig. 9 for details). In magnetic materials, *H*_c_ is proportional to the strength of PMA^49^. The larger *H*_c_ in device with double-sided AlO_x_ aligns with the finding that AlO_x_ can enhance the interfacial PMA^45–47^. In addition to PMA, a recent calculation has also suggested that bringing MnBi_2_Te_4_ surface close to a polar insulator can modify the surface potential, which is helpful for the QAH effect^30^. As a polar insulator, Al_2_O_3_ may also play an additional role in enhancing the QAH effect. It is worth noting that although all current experiments support the scenario that AlO_x_ likely contributes to magnetism, the microscopic mechanism remains inadequately understood owing to the challenges in directly measuring the interfacial magnetism. Further studies are required to elucidate the exact mechanisms.

In summary, we report the successful realization of the QAH effect in MnBi_2_Te_4_ devices capped with AlO_x_ by employing a revised fabrication method based on the standard EBL. By simply depositing an AlO_x_ layer on top of MnBi_2_Te_4_, we observe a substantial enhancement of the AH effect, ultimately achieving quantization. Our experiments resolve a longstanding challenge in the field of magnetic topological materials, paving the way for fabricating high-quality devices and investigating the intricate interplay between nontrivial topology and 2D magnetism. Recently, novel transport phenomena unavailable in previous QAH systems have already been observed in 7-SL MnBi_2_Te_4_ with current configuration, and the enhancement of surface magnetism by AlO_x_ is considered crucial to explaining these new phenomena^50^. This simple yet effective method is not only significant for fundamental studies, but also lays the groundwork for creating novel topological spintronic devices^3,43,47^. It is important to note that the current exploration on utilizing AlO_x_ to achieve the QAH effect is still in the initial stage. More refined control of the AlO_x_ growth parameters^45,46^ and the interface of oxide/MnBi_2_Te_4_ would further optimize the QAH effect, which remains a promising topic for future studies.

## Methods

### Crystal growth

High-quality MnBi_2_Te_4_ single crystals were synthesized by directly mixing Bi_2_Te_3_ and MnTe with a ratio of 1:1 in a vacuum-sealed silica ampoule. For crystal #1, the mixture was first heated up to 700 °C, and then slowly cooled down to 591 °C, followed by a long period of annealing process. The phase and crystal structure were examined by X-ray diffraction on a PANalytical Empyrean diffractometer with Cu Kα radiation. For crystal #2, a small amount of Te was added to the mixture, with the ratio between Bi_2_Te_3_, MnTe, and Te modified to 1:1:0.2. The ampoule was then slowly heated to 900 °C and maintained at this temperature for 1 h. Subsequently, it was cooled down to 700 °C, holding for 1 hour and then gradually cooled to 585 °C and maintained for 12 days. After the annealing, the ampoule was quenched in water to avoid phase impurities. Apart from devices #2, #4, #6, and #8, all other 13 devices were obtained from crystal #2. The four devices have already represented the samples showing the largest AH conductivity in the devices prepared from crystal #1.

### Device fabrication

MnBi_2_Te_4_ flakes were mechanically exfoliated onto 285 nm thick SiO_2_/Si substrate by using the Scotch tape method in an Ar-filled glovebox with O_2_ and H_2_O level lower than 0.1 ppm. Initially, the substrate was thoroughly cleaned with acetone, isopropanol, and deionized water. Then the surface of SiO_2_/Si was treated with air plasma at ~125 Pa for 3 min. The tape-covered substrate was heated up to 60 °C for 3 min to facilitate smooth exfoliation of the single crystals into flakes. Micrometer-sized thin flakes can be obtained by mechanically exfoliation on thick flakes for several times. The thickness was identified by optical contrast measurement in the glovebox immediately after exfoliation. After the target flakes were obtained, a 3-nm aluminum was deposited onto the surface using a thermal evaporator with a deposition rate 0.04 nm/s under a vacuum better than 4 × 10^−4^ Pa. Oxygen was then introduced into the chamber, and the aluminum layer was oxidized for 5 min at a pressure of 2 × 10^−2^ Pa. For device #11, an extra deposition process with longer time was employed to compare the influence of different AlO_x_ parameter on transport. During this process, an additional 30-nm AlO_x_ was deposited under a controlled oxygen environment at a pressure of 2 × 10^−2^ Pa.

To assess the effect of PMMA on the MnBi_2_Te_4_ samples, 270 nm thick PMMA was spin-coated onto the samples in an Ar-filled glovebox at a controlled speed of 4000 round/min. The samples were then heated at 60 °C for 7 min and left to stabilize in the glovebox for 24 h. Subsequently, the samples were than immersed in acetone for 20 min, rinsed with acetone followed by isopropanol, and their optical contrasts were measured immediately after the removal of PMMA. Standard EBL was employed on MnBi_2_Te_4_ samples to pattern the Hall bar structure. The oxidized aluminum was first etched from the sample surface using an Ar ion milling machine at a pressure of 2 × 10^−4^ Torr for 75 s. Cr/Au electrodes (3/50 nm) were then deposited using a thermal evaporator connected to a glovebox. Following this, the samples were again spin-coated with PMMA adopting the same parameters as before for further protection.

### Transport measurement

Standard four probe transport measurements for devices #1 to #16 were carried out in a cryostat with the lowest *T* of ~1.5 K and an out-of-plane magnetic field up to ~8 T. The longitudinal and Hall voltages were acquired simultaneously via two lock-in amplifiers with an AC current (100 nA, 13 Hz) generated by a Keithley 6221 current source meter. For device #17, the transport was performed in a dilution refrigerator with AC current excitation of 10 nA at 13 Hz. To correct the geometrical misalignment, both the longitudinal and Hall signals were symmetrized and antisymmetrized with respect to the magnetic field. The back-gate voltage was applied by a Keithley 2400 source meter through the SiO_2_/Si substrate.

### MFM measurement

Cryogenic MFM experiments were conducted in a commercial atomic force microscope (atto-AFM) equipped with commercial cantilevers (spring constant *k* ≈ 2.8 N/m and resonance frequency ≈75.8 kHz) in a closed-cycle helium cryostat. An out-of-plane magnetic field was applied using a superconducting magnet. MFM images were taken in a constant height mode with lift height of ~200 nm. The MFM signal, the change of cantilever resonance frequency, is proportional to the gradient of out-of-plane stray field. Electrostatic interaction was minimized by balancing the tip-surface potential difference.

## Supplementary information

Supplementary Information

Transparent Peer Review file

## Data Availability

All data supporting the finding in the study are presented within the main text and the supplementary information. All data are available from the corresponding author upon request.
