# Supplementary material for: Towards the quantized anomalous Hall effect in AlOx-capped MnBi2Te4
Source: Nat Commun. 2025 Feb 18;16:1727. doi: 10.1038/s41467-025-57039-7 (PMC11836109; doi:10.1038/s41467-025-57039-7)
Supplement: Supplementary file 1 — Supplementary Information [file 41467_2025_57039_MOESM1_ESM.pdf]

Supplementary Information

Towards the quantized anomalous Hall effect in AlO_x-capped $MnBi_2Te_4$

Yongqian Wang[1,2*], Bohan Fu[1,2*], Yongchao Wang[3], Zichen Lian[3], Shuai Yang[1,2], Yaoxin Li[3], Liangcai Xu[3], Zhiting Gao[4], Xiaotian Yang[5], Wenbo Wang[5], Wanjun Jiang[3,6], Jinsong Zhang[3,6,7], Yayu Wang[3,6,7,8], Chang Liu[1,2†]

[1]*School of Physics, Renmin University of China, 100872, Beijing, China*

[2]*Key Laboratory of Quantum State Construction and Manipulation (Ministry of Education), Renmin University of China, Beijing, 100872, China*

[3]*State Key Laboratory of Low Dimensional Quantum Physics, Department of Physics, Tsinghua University, Beijing 100084, China*

[4]*Beijing Academy of Quantum Information Sciences, Beijing 100193, China*

[5]*School of Physical Science and Technology, ShanghaiTech Laboratory for Topological Physics, ShanghaiTech University, 201210 Shanghai, China*

[6]*Frontier Science Center for Quantum Information, Beijing 100084, China*

[7]*Hefei National Laboratory, Hefei, 230088, China*

[8]*New Cornerstone Science Laboratory, Frontier Science Center for Quantum Information, Beijing 100084, P. R. China*

[*] These authors contributed equally to this work.

[†] Emails: liuchang_phy@ruc.edu.cn;

Contents

Supplementary Note 1:

Influence of etching on the thickness of $MnBi_2Te_4$ in the contact region

Supplementary Note 2:

Variation of optical contrast during the fabrication process

Supplementary Note 3:

Current-voltage curves of the AlO_x capping layer

Supplementary Note 4:

V_g independent magnetism in another 7-SL thick device #11

Supplementary Note 5:

Aging effect under atmospheric condition on transport properties

Supplementary Note 6:

Direct visualization of magnetism in $MnBi_2Te_4$ with and without AlO_x

Supplementary Note 7:

QAH effect in $MnBi_2Te_4$ with single and double-sided AlO_x contact

Supplementary Figure 1 to 9

Supplementary Note 1:

Influence of etching on the thickness of $MnBi_2Te_4$ in the contact region

The Ar etching process can potentially reduce the thickness of the $MnBi_2Te_4$ layer in the contact region. Through careful optimization and selection of appropriate etching parameters, this issue can be largely mitigated. To ensure the integrity of $MnBi_2Te_4$ in the contact regions, we employed both optical contrast (O_c) and atomic force microscopy measurements to characterize the thickness of $MnBi_2Te_4$ before and after the etching process. These measurements confirmed that the contact regions remained unaffected by the etching procedure.

Supplementary Figure 1 displays the optical images of two $MnBi_2Te_4$ samples that were etched under different parameters. The AlO_x on the $MnBi_2Te_4$ surface within the blue frame was subjected to the Ar ion etching, while the region outside remained unetched. Notably, for the optical image obtained using the parameters in the main text (Supplementary Figure 1a), there is no discernible variation in O_c, suggesting that the $MnBi_2Te_4$ thickness remains largely unaffected. In contrast, when the etching parameter deviates from the optimal value, such as an increased etching duration, a substantial reduction in O_c is observed, as shown in Supplementary Figure 1b. From the perspective of O_c, the transport properties of our devices are not influenced by the etching process.

To more quantitatively assess the effect of etching on $MnBi_2Te_4$ thickness, we performed atomic force microscopy measurement on the etched devices. The main results are displayed in Supplementary Figure 2. The atomic force microscopy measurement suggests that the step height at the edge of the etched regime (Supplementary Figure 2b) precisely corresponds to the thickness of the deposited AlO_x layer (Supplementary Figure 2a). Within the resolution limit of the atomic force microscopy, we did not detect significant reduction in the $MnBi_2Te_4$ thickness. These results provide more strong evidence that, under the optimized etching parameters used in our experiment, the transport properties are not influenced by changes in the thickness of the contact region.

Supplementary Note 2:

Variation of optical contrast during the fabrication process

Supplementary Figures 3a and 3b show the optical images of devices at different stages of fabrication utilizing two different techniques. In the method employed in the main text, we initially used the mechanical exfoliation method to obtain few-layer flakes on a substrate, as shown in Step 1 of Supplementary Figure 3a. This process typically yields flakes of various thicknesses. By measuring their O_c, we can identify the thickness of the flakes and selected the target flake. In the second step, we deposited a 3-nm Al capping layer on the top surface by thermal evaporation. The Al was oxidized in an O_2 environment to form a compact AlO_x layer. In the third step, a sharp needle was used under a microscope to remove the thick flakes surrounding the target flake, ensuring sufficient space for the Cr/Au electrodes. In the next, we spin-coated a PMMA layer on the sample surface and performed a standard electron beam lithography to define the designed Hall bar structure. The target regimes of the pattern were developed in the fourth step, showing the position of the electrodes, as indicated by the red dashed frames. Excess AlO_x was then removed using argon ion etching to expose the underlying $MnBi_2Te_4$. Finally, 3/50 nm Cr/Au electrodes were deposited through thermal evaporation, as illustrated in Step 5. For comparison, Supplementary Figure 3b displays the images of the device fabrication process without AlO_x. O_c analysis clearly suggests the contrast value decreases immediately after the device contacts PMMA in the third step. The variation of O_c in each step is shown in Supplementary Figure 3c.

Supplementary Note 3:

Current-voltage curves of the AlO_x capping layer

To eliminate the potential shunting effects caused by incomplete oxidation of AlO_x layer, we conducted two-terminal transport measurements in devices with different configurations. As shown in Supplementary Figure 4a, in the first configuration, we fabricated a $MnBi_2Te_4$ device with Cr/Au electrodes deposited directly on the 3-nm AlO_x layer without undergoing Ar ion etching. This configuration allows for examining the conductivity of AlO_x layer in the vertical direction. Supplementary Figure 4b shows the current-voltage (*I-V*) curve measured at zero magnetic field ($\mu_0 H = 0$) and room temperature (T). The *I-V* curve manifests a pronounced nonlinear behavior with increasing V to above 0.6 V, indicating that the AlO_x formed a good insulating layer between the Cr/Au electrodes and the sample. At low V regime, the resistance estimated by the linear *I-V* behavior exceeds 20 MΩ, a value three orders of magnitude higher than that of the typical resistance of a 7-SL $MnBi_2Te_4$ device. This result suggests the high insulation of a 3 nm AlO_x layer along the z direction.

To further demonstrate the insulating properties of the AlO_x layer, we fabricated another device with only a 3-nm AlO_x layer deposited on the substrate. The optical image and schematic side view are shown in Supplementary Figure 4c. By measuring the two-terminal resistance, one can assess the conductivity of AlO_x in the lateral direction. The *I-V* curve presented in Supplementary Figure 4d exhibits more pronounced insulating properties than that in the vertical direction. Within the V range of ±10 V, the two-terminal resistance estimated from the linear *I-V* curve in the low V range has exceeded 10 GΩ, far surpassing the typical resistance of $MnBi_2Te_4$ flakes. These results undoubtedly demonstrate the highly insulating properties of AlO_x layer in our experiments, ensuring that it does not contribute to any electrical signal during the transport measurements shown in the main text.

Supplementary Note 4:

V_g independent magnetism in another 7-SL device #11

Supplementary Figures 5a and 5b show the μ_0H dependent σ_{xy} and σ_{xx} for another 7-SL device #11 at varied V_g and Ts. In comparison to the device #16 situated on the same substrate, device #11 does not achieve full quantization at $\mu_0H = 0$, with σ_{xy} only reaching ~ 0.4 e^2/h. Besides, the plateau-to-plateau transition is also more gradual in device #11, paralleled with a weaker double-peak feature in σ_{xx}. These characteristics indicate that the AFM order in this device may not be as strong as that in device #16. Nonetheless, this sample exhibits overall similar V_g independent magnetic properties.

As presented in Supplementary Figure 5c, we first performed a critical behavior fitting of H_c as varying T. The fitting results largely recapitulate the main characteristics presented in the main text for device #16 and agree with previous reflective magnetic circular dichroism (RMCD) measurements in $MnBi_2Te_4$ flakes, which suggests a critical exponent (β) between 0.47 and 0.54 for samples thicker than five SLs[1]. Notably, at Ts below 10 K, the data points for all V_gs deviate from the fitted curves. Similar phenomena were also observed in previous neutron diffraction experiments[2], where the experimentally derived β was approximately 0.50 near T_N, but is decreased to between 0.32 and 0.35 at lower Ts. Considering that β characterizes the critical behaviors near the magnetic phase transition, high-T fittings close to T_N are deemed more accurate. The mechanism behind the deviation at low Ts requires more further investigation. Supplementary Figure 5d presents the evolution of fitting results as varying V_g, where β remains approximately at ~ 0.54, and T_N remains around ~ 21.4 K. Both results are highly consistent with the data of device #16 in the main text. In Supplementary Figure 5e, the colormap of σ_{xy} plotted against μ_0H and V_g shows a horizontal boundary between the blue and orange regions, indicating that H_c remains almost unchanged irrespective of the change in V_g. These observations further support the V_g independent antiferromagnetism in odd-SL $MnBi_2Te_4$.

To further study the V_g dependent magnetism, we measured the transport properties in a low-quality device #10 without AlO_x. For the 7-SL device #10 without AlO_x capping layer, the magnetic properties exhibit similar behaviors to those of devices with AlO_x capping layer

(Fig. 4 and Supplementary Figure 5). H_c also exhibits V_g independent behaviors, and the T-dependent scaling analysis of H_c reveals a critical exponent $\beta \sim 0.54$, consistent with $\beta \sim 0.52$ and 0.54 for the two high-quality devices with AlO_x capping layer. However, the fitted $T_N \sim$ 20.0 K is slightly lower, suggesting weaker magnetism likely owing to the absence of AlO_x. These consistent behaviors further support the conclusion in the manuscript. The main results are presented in Supplementary Figure 6.

Supplementary Note 5:

Aging effect under atmospheric condition on transport properties

In the QAH effect, in addition to the potential influence of fabrication on device quality, the aging effect in various environments can also destroy the quantized transport behaviors. Particularly, when devices must be transferred between different systems, exposure to air is likely to change the electronic and magnetic properties. In this section, we discuss how aging effect in air affects the quality of our device.

Supplementary Figure 7a displays the phase diagram of Hall resistivity ρ_{yx} as a function of μ_0H and V_g for device #16. With μ_0H switches the magnetic configuration from AFM to FM order, the V_g range for the QAH state or Chern insulator state is increased from 5 V to 30 V. Concurrently, with ρ_{yx} approaches quantization, the longitudinal resistivity ρ_{xx} is significantly reduced to a small value in high μ_0H, suggesting that the transport is governed by 1D dissipationless chiral edge states (Supplementary Figure 7b). Interestingly, a sudden jump is observed at $\mu_0H = 2.2$ T in both ρ_{yx} and ρ_{xx}. Given that the QAH effect arises from the magnetic exchange gap on the topological surface states, this sudden jump indicates a magnetic phase transition associated with spin flip or flop at the surface or subsurface. A more detailed discussion on the quantum phase transition in $MnBi_2Te_4$ QAH state can be found in our recent experiment[3]. Supplementary Figure 7c shows the phase diagram of the Hall angle defined by ρ_{yx}/ρ_{xx}. The magenta dashed line denotes the position of Hall angle of 45° ($\rho_{yx}/\rho_{xx} = 1$). The overall regime where Hall angle exceeds 45° overlaps well with the quantization area in ρ_{yx} colormap.

Previous studies on the QAH effect in molecular beam epitaxy grown magnetic TIs have demonstrated that an AlO_x layer can effectively preserve the sample quality[4,5]. To explore the protective effects of AlO_x on exfoliated $MnBi_2Te_4$, we analyze the transport properties of the sample before and after undergoing a thermal cycling process and exposure to the air for five minutes. The main results are presented in Supplementary Figure 7d to 7f. Interestingly, we observe a shift of the charge neutral point from 10 V to 5 V after re-cooling, indicating electron-type doping during the process of the thermal cycling and air exposure. At low μ_0H side, there is a noticeable decrease in ρ_{yx}, and the extent of the blue region in Supplementary Figure

7a is reduced, as displayed in Supplementary Figure 7d. In the high-field regime, only a shift of V_g is observed, with no significant reduction in the quantization region. This phenomenon suggests that aging effect likely influences the surface electronic structure via reducing the surface perpendicular magnetic anisotropy in the AFM state, thereby decreasing the exchange gap near $\mu_0 H = 0$. However, at high $\mu_0 H$, all the magnetic moments are aligned by the external field along one direction, maintaining the overall magnetization unchanged. Therefore, there are minimal changes to the exchange gap. The changes of charge neutral point and the reduction of quantization are also evident in the phase diagrams of ρ_{xx} and ρ_{yx}/ρ_{xx}, as displayed in Supplementary Figures 7e and 7f.

These experimental findings highlight that, although AlO_x is essential for achieving the QAH effect, $MnBi_2Te_4$ remains vulnerable to the process of thermal cycling and air exposure. Possible reasons include thermal expansion and contraction during the thermal cycling process, which may lead to cracks on the surface of the AlO_x thin layer. This process will allow air to penetrate through the capping layer and directly contact the sample. Therefore, further enhancement of the AlO_x layer quality to improve its protective effect on $MnBi_2Te_4$ warrants future investigation.

Supplementary Note 6:

Direct visualization of magnetism in $MnBi_2Te_4$ with and without AlO_x

Supplementary Figure 8 displays the magnetic force microscopy (MFM) measurement results obtained from two different regions of the same 7-SL $MnBi_2Te_4$ flake. Although MFM cannot directly measure the absolute magnetization, the magnetic contrast signals at the spin flipping region (coercive field) allow us to determine the relative strength of the magnetism. Similar technique has already been employed in our previous studies on the ferromagnetism of Cr/V-doped $(Bi,Sb)_2Te_3$ QAH system[6]. Remarkably, our new MFM results align well with our expectation. We first exfoliated a 7-SL flake using the mechanical cleavage method. Then, a part of the flake was covered with PDMS. An AlO_x capping layer was then deposited over the entire surface. After that, we removed the PDMS layer and employed a sharp needle to divide the sample into separate parts with and without AlO_x. This process enables the direct comparison of the influence of AlO_x on magnetism in the same sample. Supplementary Figure 8 presents the imaging results obtained from two distinct areas of the 7-SL flake. It clearly shows that the region with AlO_x exhibits a stronger magnetic contrast signal than that without AlO_x capping layer. At $\mu_0 H$ where the magnetic configuration switches from ↓↑↓↑↓↑↓ (blue) to ↑↓↑↓↑↓↑ (red) state, where ↓ and ↑ represents the down and up magnetization of each layer, a pronounced magnetic contrast signal is observed in the AlO_x capped part. Conversely, the contrast signal is significantly weaker in the area without AlO_x capping. The diminished magnetism even hinders the stable presence of downward magnetization within the domain flipping region, making it difficult to observe a clear contrast signal (blue). Notably, in previous experiments, even in $MnBi_2Te_4$ exfoliated in ultra-high vacuum, the gap was not observed[7]. It is believed that the ordered spin state in the bulk may display fragility at the surface layers. Our imaging result aligns with these studies.

Supplementary Note 7:

QAH effect in $MnBi_2Te_4$ with single and double-sided AlO_x contact

In addition to MFM imaging, we have also conducted transport measurements to study the properties of $MnBi_2Te_4$ flake with both surfaces contacted with AlO_x. Before mechanical exfoliation, we deposited a 3-nm AlO_x on the bottom surface of $MnBi_2Te_4$. Then, we cleaved the crystal onto a Si/SiO_2 substrate and deposited an AlO_x capping layer on the surface using the parameters in the main text. If AlO_x indeed plays a role in enhancing the interfacial perpendicular magnetic anisotropy, as demonstrated in previous studies[8,9], one would expect that having both surfaces in contact with AlO_x would further enhance the magnetic anisotropy of the sample. For magnetic materials, H_c is proportional to the strength of perpendicular magnetic anisotropy[10]. Therefore, by comparing the value of H_c in devices with single-sided and double-sided AlO_x contact, we can infer the influence of AlO_x on the magnetic properties of $MnBi_2Te_4$. As shown in Supplementary Figure 9, our transport results are consistent with this expectation. For the device where both surfaces are in contact with AlO_x, we observe a notable enhancement in its H_c compared with the device with only top surface AlO_x contact. This result is consistent with our hypothesis that AlO_x may contribute to the enhancement of magnetism in $MnBi_2Te_4$.

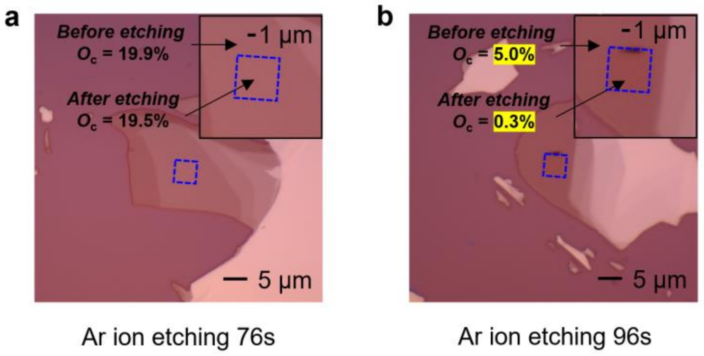

Supplementary Figure 1 | Optical images of $MnBi_2Te_4$ etched with different etching parameters. a, Image of $MnBi_2Te_4$ flakes using the parameters shown in the main text. **b,** Image of $MnBi_2Te_4$ flakes with longer etching time. The blue area indicates the etched region.

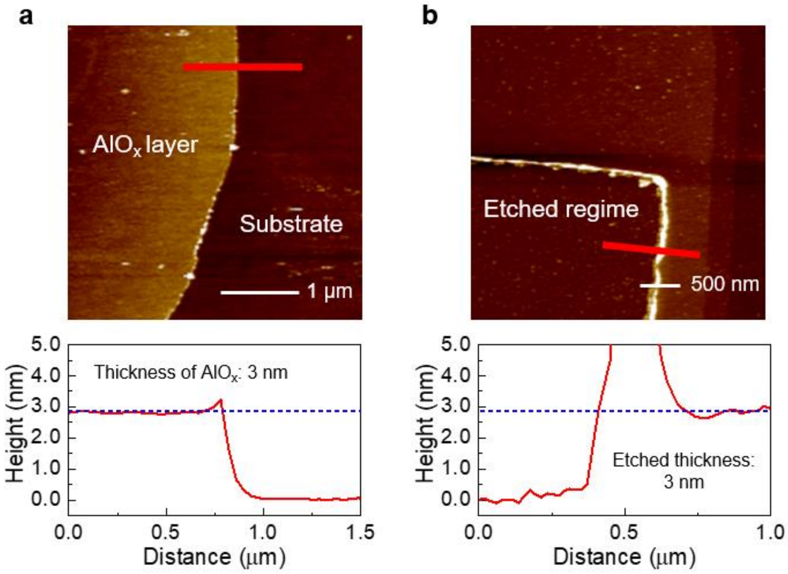

Supplementary Figure 2 | Atomic force microscopy results of the thickness of AlO_x layer and the step height near the etched region. a, Morphology of AlO_x and height profile along the red line. The thickness of AlO_x layer is ~ 3 nm. **b,** Morphology and the step height near the etched region. The height near the boundary of the etched region is also ~ 3 nm, consistent with the thickness of the AlO_x layer.

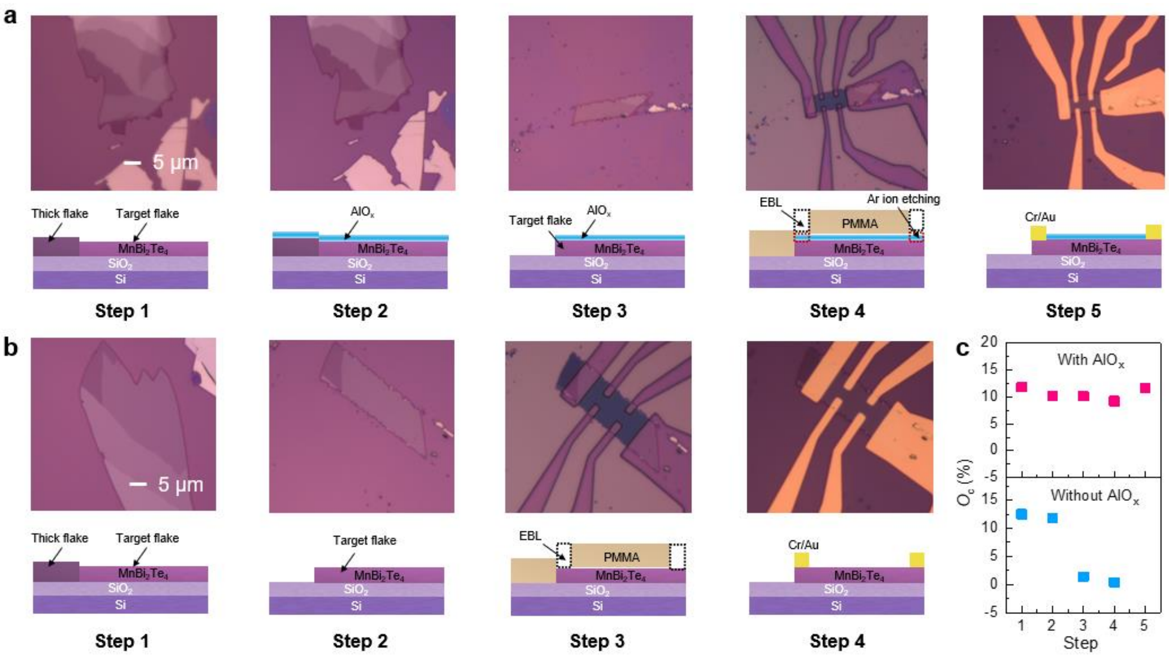

Supplementary Figure 3 | Optical images of two 7-SL $MnBi_2Te_4$ devices taken at different steps of the fabrication. a, Optical images of a 7-SL device fabricated by the revised method. The bottom panel displays the side view of the device in different fabrication steps. **b,** Optical images of another 7-SL device fabricated by standard electron beam lithography. **c,** O_c variation in different steps of the two methods.

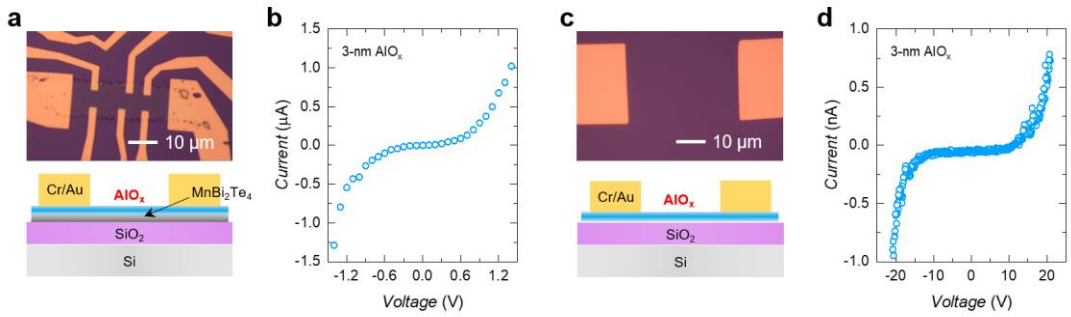

Supplementary Figure 4 | Current-voltage curves of the AlO_x layer in two different configurations. a, Optical image and schematic side view of a 7-SL device with electrodes directly deposited on the AlO_x layer. **b,** Two-terminal *I*-*V* curve measured at $\mu_0 H = 0$ and room *T*. **c,** Optical image and schematic side view of a 3-nm thick AlO_x device. **d,** Two-terminal *I*-*V* curve measured between ± 20 V.

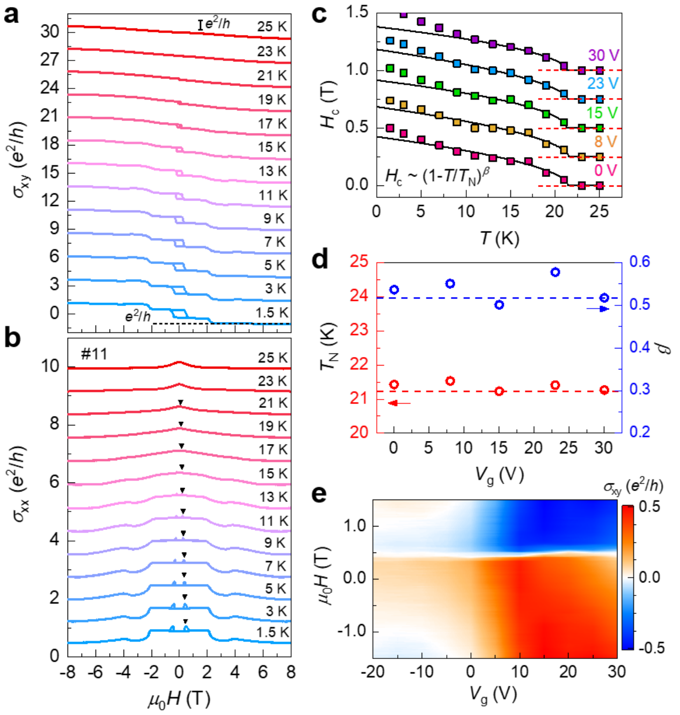

Supplementary Figure 5 | Transport and magnetic properties tuned by V_g. a-b, Dependence of σ_{xy} and σ_{xx} on μ_0H near the charge neutral point (CNP) for varied Ts. **c,** H_c extracted from the field sweeping data plotted as a function of T for various V_gs. Data points are marked by solid squares, with the black lines representing the power-law fitting ~ $(1-T/T_N)^\beta$. **d,** Variation of T_N and β as V_g. **e,** Colormap of σ_{xy} in the parameter space of μ_0H and V_g. The white horizontal boundary between blue and orange region indicates the V_g independent behavior of H_c.

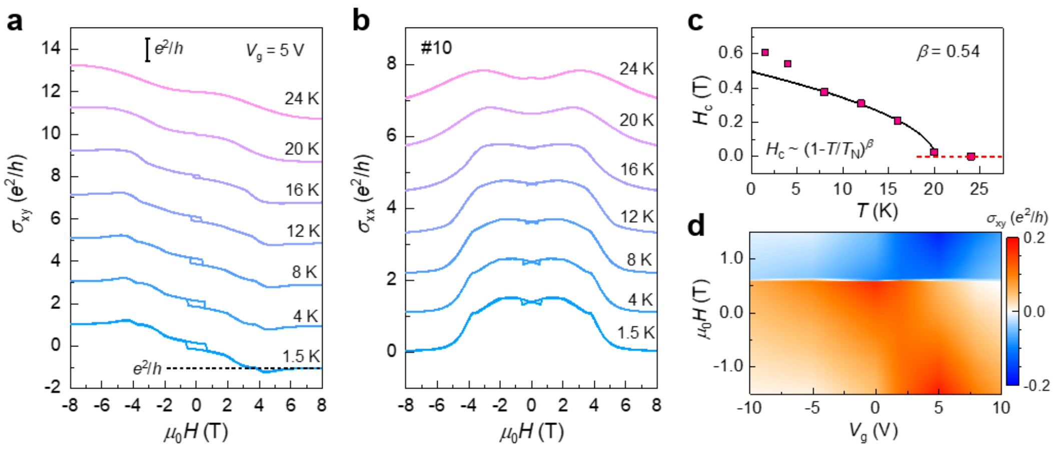

Supplementary Figure 6 | Magnetic field dependence of the transport behaviors of a 7-SL device without AlO_x at different V_g and Ts. a-b, μ_0H dependent σ_{xy} and σ_{xx} at the CNP for varied Ts. **c,** H_c extracted from the field sweep data as a function of T. The black lines are the fittings in the form of $(1-T/T_N)^\beta$. **d,** Colormap of σ_{xy} in the parameter space of μ_0H and V_g.

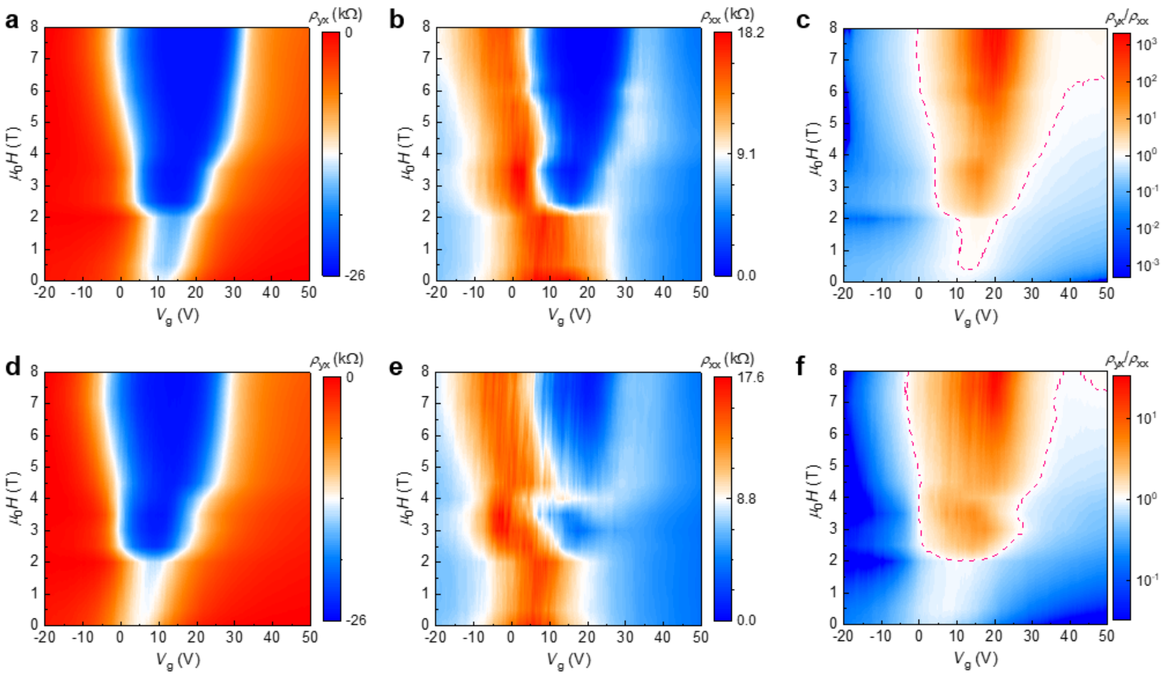

Supplementary Figure 7 | Transport properties and protective role of AlO_x layer on exfoliated $MnBi_2Te_4$. a-c, Colormaps of ρ_{yx}, ρ_{xx}, and ρ_{yx}/ρ_{xx} in the parameter space of μ_0H and V_g at $T = 1.5$ K. **d-f**. Colormaps of ρ_{yx}, ρ_{xx}, and ρ_{yx}/ρ_{xx} of the device after undergoing a thermal cycling process and exposure to air for five minutes.

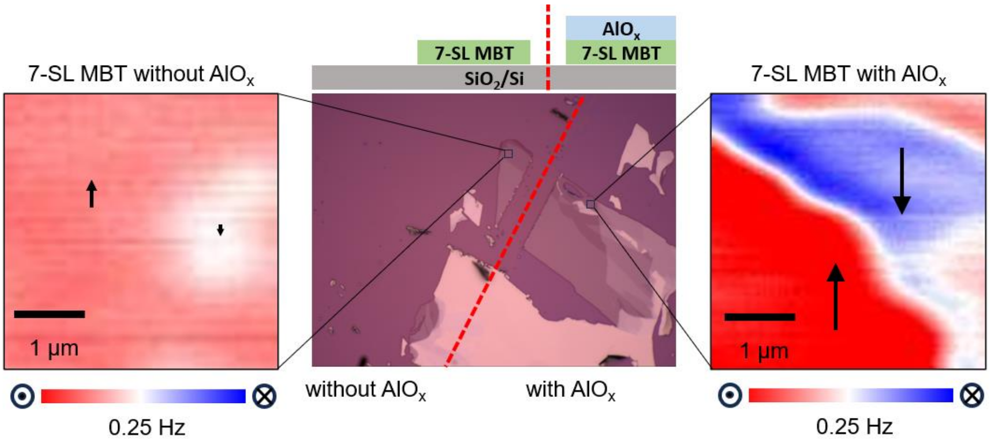

Supplementary Figure 8 | MFM results obtained at the H_c across two distinct parts with and without AlO_x in the same 7-SL $MnBi_2Te_4$. The blue and red color denotes the direction of downward and upward magnetization.

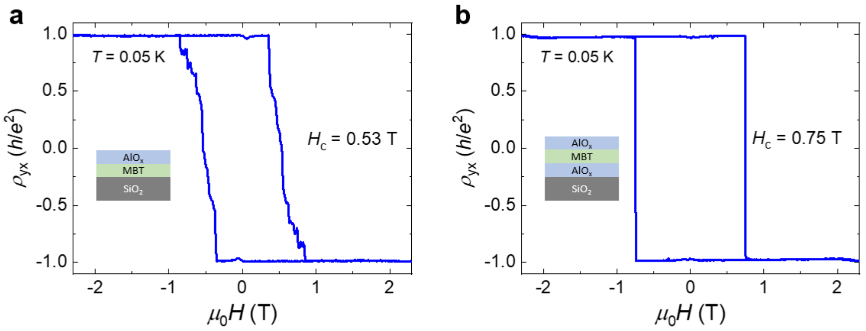

Supplementary Figure 9 | QAH effect in two $MnBi_2Te_4$ flakes with different configurations. a, QAH effect in $MnBi_2Te_4$ device with single-sided AlO_x. **b,** QAH effect in $MnBi_2Te_4$ device with double-sided AlO_x. Both samples exhibit full quantization at zero magnetic field.

Supplementary References

1. Yang S. Q., Xu X. L., Zhu Y. Z.*, et al.* Odd-Even Layer-Number Effect and Layer-Dependent Magnetic Phase Diagrams in $MnBi_2Te_4$. *Phys. Rev. X*, **11**, 011003 (2021).

2. Ding L., Hu C. W., Ye F., Feng E. X., Ni N., Cao H. B. Crystal and magnetic structures of magnetic topological insulators $MnBi_2Te_4$ and $MnBi_4Te_7$. *Phys. Rev. B*, **101**, 020412(R) (2020).

3. Lian Z., Wang Y., Wang Y.*, et al.* Antiferromagnetic Quantum Anomalous Hall Effect Modulated by Spin Flips and Flops. p. arXiv:2405.08686; 2024.

4. Gao Z., Guo M., Lian Z.*, et al.* Low-damage photolithography for magnetically doped $(Bi,Sb)_2Te_3$ quantum anomalous Hall thin films. *Chin. Phys. B*, **32**, 117303 (2023).

5. Tay H., Zhao Y.-F., Zhou L.-J., Zhang R., Yan Z.-J., Zhuo D., Chan M. H. W., Chang C.-Z. Environmental Doping-Induced Degradation of the Quantum Anomalous Hall Insulators. *Nano Lett*, **23**, 1093-1099 (2023).

6. Wang W. B., Ou Y. B., Liu C., Wang Y. Y., He K., Xue Q. K., Wu W. D. Direct evidence of ferromagnetism in a quantum anomalous Hall system. *Nat. Phys.*, **14**, 791-795 (2018).

7. Hao Y. J., Liu P. F., Feng Y.*, et al.* Gapless Surface Dirac Cone in Antiferromagnetic Topological Insulator $MnBi_2Te_4$. *Phys. Rev. X*, **9**, 041038 (2019).

8. Dieny B., Chshiev M. Perpendicular magnetic anisotropy at transition metal/oxide interfaces and applications. *Rev. Mod. Phys.*, **89**, 025008 (2017).

9. Hellman F., Hoffmann A., Tserkovnyak Y.*, et al.* Interface-induced phenomena in magnetism. *Rev. Mod. Phys.*, **89**, 025006 (2017).

10. Coey J. M. D. *Magnetism and magnetic materials*. Cambridge University Press: Cambridge, 2010.
